# Extracorporeal Membrane Oxygenation in COVID-19 Treatment: a Systematic Literature Review

**DOI:** 10.21470/1678-9741-2020-0397

**Published:** 2021

**Authors:** Tatiana Farias de Oliveira, Carlos Alberto de Oliveira Rocha, Aisla Graciele Galdino dos Santos, Luiz Carlos Francelino Silva Junior, Saulo Henrique Salgueiro de Aquino, Euclides José Oliveira da Cunha, Rafaela Campos Alcântara, Rodrigo da Rosa Mesquita, Gabriel Monteiro Arnozo, Fernanda Mayara Santos Santana, Etvaldo Rodrigues da Silva Filho, Carlos Dornels Freire de Souza

**Affiliations:** 1 Department of Medicine, Universidade Federal de Alagoas, Arapiraca, Alagoas, Brazil.

**Keywords:** Extracorporeal Membrane Oxygenation, Coronavirus Infections, COVID-19, Patient Discharge

## Abstract

**Introduction:**

The present study intends to systematically review the literature on the use of extracorporeal membrane oxygenation (ECMO) in patients with coronavirus disease 2019 (COVID-19).

**Methods:**

The research was carried out according to the recommendations of the Preferred Reporting Items for Systematic Reviews and Meta-Analyzes (PRISMA). Studies were selected from PubMed/MEDLINE and LILACS databases between December 2019 and May 17 2020, using the descriptors "ECMO AND COVID-19", "Extracorporeal Membrane Oxygenation AND COVID-19", "ECLS AND COVID-19", and "Extracorporeal Life Support AND COVID-19". Exclusion criteria were government epidemiological bulletins, comments, literature reviews, and articles without full access to content.

**Results:**

Two hundred and thirty-three scientific productions were found, however only 18 did not met the exclusion criteria and could be included in this study, amouting to a total of 911 patients - 624 (68.5%) men, 261 (28.6%) women, and 26 (2.8%) without sex information. The mean age of the patients was 53.7 years. ECMO was necessary in 274 (30.1%) people (200 [73%] submitted to veno-venous ECMO, nine [3.3%] to veno-arterial ECMO, and seven [2.5%] moved between these two types or needed a more specific ECMO according to the disease prognosis). Five studies did not specify the type of ECMO used, amounting 57 (20.8%) patients. Five patients (1.8%) were discharged, 77 (28.1%) died, 125 (45.6%) remained hospitalized until publication time of their respective studies, and 67 patients (24.4%) had no outcome information.

**Conclusion:**

It is evident that more research, covering larger populations, must be carried out in order to clearly elucidate the role of ECMO in the treatment of COVID-19.

**Table:** 

Abbreviations, acronyms & symbols	
**ARDS**	**= Acute respiratory distress syndrome**
**COVID-19**	**= Coronavirus disease 2019**
**ECMO**	**= Extracorporeal membrane oxygenation**
**FiO2**	**= Fraction of inspired oxygen**
**PaO2**	**= Partial pressure of oxygen**
**SARS-CoV-2**	**= Severe acute respiratory syndrome coronavirus 2**
**USA**	**= United States of America**
**VA-ECMO**	**= Veno-arterial extracorporeal membrane oxygenation**
**VAV-ECMO**	**= Veno-arterio-venous extracorporeal membrane oxygenation**
**VV-ECMO**	**= Veno-venous extracorporeal membrane oxygenation**
**VVA-ECMO**	**= Veno-veno-arterial extracorporeal membrane oxygenation**
**VVV-ECMO**	**= Veno-veno-venous extracorporeal membrane oxygenation**
**WHO**	**= World Health Organization**

## INTRODUCTION

Coronavirus disease 2019 (COVID-19), a disease caused by a new type of coronavirus, the severe acute respiratory syndrome coronavirus 2 (SARS-CoV-2), is being considered cause of the most important health crisis of the last hundred years^[1]^. Originating in the People's Republic of China, the disease quickly spread to all continents. On March 11 2020, the World Health Organization (WHO) declared a pandemic status ^[2]^.

In June 12 2020, more than 7.4 million people were infected and 400,000 deaths had occurred from the disease worldwide. Behind the United States of America (USA) alone, with two million cases, Brazil occupied, on that date, the second position in the ranking of countries with more cases of COVID-19, with about 850 thousand cases. In terms of number of deaths, USA remains in the lead, accounting for 115,000 deaths^[3]^.

SARS-CoV-2 infection can lead to the development of acute respiratory distress syndrome (ARDS)^[4]^. Approximately 14% of COVID-19 cases are serious and 5% are critical^[5]^. In such cases, therapy includes protective pulmonary mechanical ventilation, neuromuscular blockade, higher positive end-expiratory pressure, pulmonary recruitment techniques, and prone positioning. When conventional therapy fails, extracorporeal membrane oxygenation (ECMO) can be considered as an alternative in certain patients^[6]^.

In ECMO treatment, there are two basic methods that can be used: veno-venous (VV-ECMO) or veno-arterial (VA-ECMO)^[7]^. Regarding to COVID-19 respiratory complications, VV-ECMO is the recommended form^[6]^. Thus, this study aims to review the literature on the use of this therapeutic strategy in patients with COVID-19.

## METHODS

### Data Sources and Search Strategies

This is a systematic review conducted according to the Preferred Reporting Items for Systematic Reviews and Meta-Analyzes, or PRISMA^[8]^, recommendations.

For this review, studies involving patients with COVID-19 were used. Studies published in the PubMed/MEDLINE and LILACS databases were screened between December 2019 and May 17 2020, using the descriptors "ECMO AND COVID-19", "Extracorporeal Membrane Oxygenation AND COVID-19", "ECLS AND COVID-19", and "Extracorporeal Life Support AND COVID-19". In addition, a manual search was carried out for references cited in the articles.

### Research Variables

The following variables were researched: country of study, study population, sex (male and female), use of ECMO, type of ECMO used in the treatment of patients with COVID-19, main outcomes, and conclusions/recommendations.

### Eligibility criteria

We included letters to the editor, clinical trials, cohorts, cross-sectional studies, clinical cases, and case series studies (published and pre-print). Government epidemiological bulletins, comments, literature reviews, articles without full access to content, and studies in animals were excluded.

### Selection of Studies

The search was carried out by four independent researchers. After this stage, three researchers independently performed the following steps: 1) reading the title and summary to identify potential eligible studies; 2) reading the full text; 3) collecting ECMO data and setting up the database. The analysis was independently conducted by two other researchers. The divergences were analyzed and resolved by consensus.

The included studies were submitted to a qualitative analysis using the Risk Of Bias In Non-randomized Studies of Interventions, or ROBINS-I^[9]^, which is a recommended tool to assess the risk of bias in non-randomized studies included in systematic reviews.

### Data Extraction

For data extraction, the researchers created a database. At this stage, the database was mounted. The data was entered by a first investigator and subsequently checked by a second investigator on the team. The systematization/analysis of the data was conducted by two other independent researchers.

## RESULTS

**Fig. 1:**
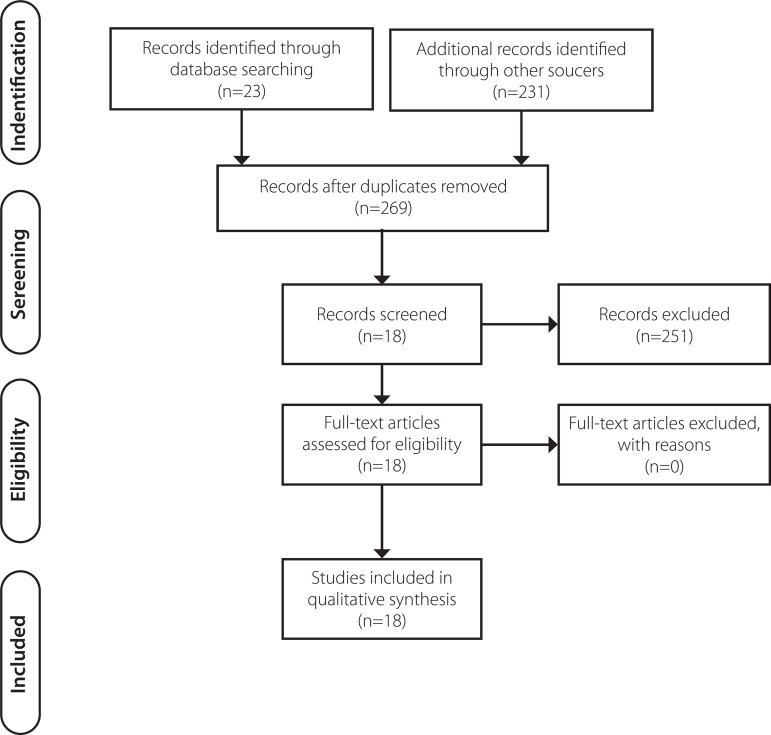
Flowchart of studies selection, 2020.

Initially, 233 scientific productions were found in the researched databases. After the successive stages of analysis, only 18 articles fit the objective of this study, pointing to ECMO as one of the resources in the treatment of COVID-19 (Figure 1): Europe^[4]^ (1 study, 333 patients), People's Republic of China^[2,6,10,11,12]^ (5 studies, 385 patients), Japan^[13,14,15,16]^ (4 studies, 98 patients), USA^[17,18,19]^ (3 studies, 43 patients), Spain^[20]^ (1 study, 48 patients), Italy^[21]^ (1 study, 1591 patients), and Switzerland^[22]^ (1 study, 1 patient).

The surveys total 911 patients confirmed with COVID-19, 68.5% (n=624) males and 28.6% (n=261) females; there was no sex information about 2.8% (n=26) of the patients. The mean age of patients in studies ^[1,2,4,6,10,11,12,13,14,15,16,17,19,21,22,26]^that reported this data was 53,7 years. and Japan was the country with the oldest patients, with the highest reported age being 81 years and the lowest, 16 years.

ECMO was necessary as a resource in those patients whose health status was severe/critical, totaling 274 (30.1%) people (Table 1). Regarding the type of ECMO, 73% (n=200) of the patients underwent VV-ECMO. Among the others, nine individuals (3.3%) required VA-ECMO and seven patients (2.5%) moved from one type to another according to the disease prognosis, or needed a more specific type of ECMO such as veno-veno-venous ECMO, veno-arterio-venous ECMO, or veno-veno-arterial ECMO. Five studies^[2,8,12,13,10]^ did not specify the type of ECMO used to treat their patients (57 [20.8%] patients) (Table 1).

**Table 1:** Characterization of the articles included in the study (n=18), 2020.

Study	Total N	Age (years)	Sex		Use of ECMO	Type of ECMO	Main outcome	Conclusion/Suggestions
			Men	Women				
Hartman ME et al.^[1]^	1	44	1 (100%)	-	1 (100%)	VV-ECMO	Hospital discharge.	The study suggests caution with the applicability of ECMO based on the experience of a single patient.
Case report								
Zeng Y et al.^[2]^	12	Mean age: 50.9 (35-76)	11 (91.7%)	1 (8.3%)	12 (100%)	Not specified	3 (25%) patients evolved without ECMO; 4 (33.3%) patients still alive with ECMO, but 2 in a coma; 5 (41.7%) patients died.	The paper suggests further studies on the use of ECMO in patients with COVID-19, and caution is needed to recommend ECMO to patients with COVID-19 in critical condition
Case series								
Marullo A et al.^[4]^	333	Mean age: 51.8	-	48 (14.4%)	157 (47.1%)	(149) VV-ECMO; (5) VA-ECMO; (2) VAV-ECMO; (1) VVV-ECMO	54 patients evolved with weaning from ECMO (18.1%)*, 57 (17.1%) patients died, the outcomes of the other patients were not reported.	The study suggests international validation of its findings and further studies on the topic.
Retrospective analysis		Median age: 54 (16-74)						
Li X et al.^[6]^	8	Mean age: 64,25	6 (75%)	2 (25%)	8 (100%)	(7) VV-ECMO and (1) VA-ECMO	3 (37.5%) patients evolved with improvement, 1 (12.5%) patient remained on mechanical ventilation, and 4 (50%) patients died.	The study reports that support for oxygenation by extracorporeal membrane can be an integral part of the critical care provided to patients with COVID-19 in centers with advanced knowledge in ECMO.
Case series		Median age: 64,5 (25-81)						
Wang D et al.^[10]^	138	Median age: 56 (22-92)	75 (54.3%)	63 (45.7%)	4 (2.9%)	Not specified	-	-
Case series								
Jacobs JP et al.^[17]^	32	Mean and median age: 52.41	22 (68.8%)	10 (31.2%)	32 (100%)	(25) VV-ECMO; (3) VA-ECMO; (1) VAV to VV-ECMO; (1) VV to VVA-ECMO; (1) VV to VVV-ECMO; (1) not specified	17 (53.12%) patients remain on ECMO, 10 (31.25%) patients died before or shortly after decannulation, and 5 (15.62%) patients are alive and extubated after ECMO removal, with 1 (3.12%) patient discharged from hospital.	The study states that their data can help define the best strategies to care for these patients and provide a framework for future research on the use of ECMO to treat patients with COVID-19.
Real-time cohort								
Taniguchi H et al.^[13]^	1	72	-	1 (100%)	1 (100%)	VV-ECMO	The patient spent 6 days on ECMO treatment (on the 12^th^ day of hospitalization), with an improvement in chest radiography. The respirator was removed after tracheostomy on the 19^th^ day of hospitalization.	The treatment of severe pneumonia in COVID-19 by ECMO must recognize pulmonary plasticity, considering the time for the introduction of ECMO and interstitial biomarkers.
Case report								
Barrasa H et al.^[20]^	48	Median age: 63 (51-75)	27 (56.3%)	21 (43.7%)	1 (2.1%)	VV-ECMO	-	The study suggests that the correct oxygenation saves lives and that the clinical observations provide useful information that can help improve management and results.
Case series								
Sultan I et al.^[18]^	10	31-62	7 (70%)	3 (30%)	10 (100%)	VV-ECMO	2 (20%) patients were successfully released from ECMO support, 1 (10%) patient is currently undergoing weaning, and 1 patient (10%) died after 9 days of ECMO due to multiorgan dysfunction. All other patients remain on ECMO.	The paper reports that its data can guide intensive care management and resource allocation of the intensive care unit and ECMO infrastructure, in addition to being an attempt to characterize the patient population using ECMO to help establish selection criteria.
Case series								
Firstenberg MS et al.^[19]^	1	51	-	1 (100%)	1 (100%)	VV-ECMO	Patient was extubated after 11 days of treatment, being discharged after other 11 days of extubation.	The study suggests timely referral to a tertiary center with established experience and standardized ECMO protocols, if it is considered a treatment for COVID-19. It also suggests future prospective multicenter studies to validate its findings in a larger cohort of patients.
Case report								
Zhan WQ et al.^[11]^	1	54	1 (100%)	-	1 (100%)	VV-ECMO	Patient left ECMO 5 days after treatment with normal vital signs, but remained on mechanical ventilation for another 10 days. The patient received oxygen inhalation for another 6 days, being discharged from the hospital on February 24.	The study strongly recommends ECMO treatment since the beginning of the illness in critical patients with COVID-19 and warns that the patient's clotting function and blood gases need to be monitored regularly to decide how long to use ECMO.
Case report								
Nakamura K et al.^[14]^	1	45	1 (100%)	-	1 (100%)	Not specified	The patient was decannulated after 11 days using ECMO and was discharged 12 days later.	-
Case report								
Bemtgen et al.^[26]^	1	52	1 (100%)	-	1 (100%)	VA-VV ECMO	ECMO continued to function, even after 24 days of treatment.	-
Case report								
Giani et al.^[21]^	1	66	1 (100%)	-	1 (100%)	VV-ECMO	-	-
Case report								
Japan ECMsOne.^[15]^	26	Mean age: 71 (45-81)**	-	-	26 (100%)	Not specified	16 (62%) patients were weaned, 6 (26%) patients were extubated and referred for rehabilitation, and 10 (38%) patients remained on ECMO. The available data from the first 14 cases demonstrated that the median number of days between intubation and ECMO was 3 days (range 0–9 days).	The study concludes that patients who presented a preserved lung compliance phenotype were probably favored by the use of ECMO and indicates the adoption of a real-time discussion platform to guide the use of ECMO. Finally, it is suggested further research to classify the ideal use of ECMO in patients with COVID-19.
Cross-sectional study								
Kato et al.^[16]^	70	Mean age: 67 (54-72)	47 (67.1%)	23 (32.9%)	2 (2.85%)	VV-ECMO	Patients were successfully treated and survived at the end of the observation period: 1 patient was extubated on day 13 of ventilation and the other one was intubated for 23 days using VV-ECMO.	-
Case series								
Yu et al.^[12]^	226	Mean age: 64 (57-70)	139 (61.5%)	87 (38.5%)	14 (6.2%)	Not specified	-	-
Cross-sectional study								
Schmiady et al.^[22]^	1	54	-	1 (100%)	1 (100%)	VV-ECMO	-	-
Case series								

COVID-19=coronavirus disease 2019; ECMO=extracorporeal membrane oxygenation; VA-ECMO=veno-arterial ECMO; VAV-ECMO=veno-arterio-venous ECMO; VV-ECMO=veno-venous ECMO; VVA-ECMO=veno-veno-arterial ECMO; VVV-ECMO=veno-veno-venous ECMO

* The study brings these adjusted data for smaller denominators due to incomplete reports.

** The study brings the available data from the first 14 cases.

Regarding the outcome related to the use of ECMO, concerning the 274 patients who used the therapeutic resource, five (n=1.8%) were discharged, 125 (45.6%) remained hospitalized until the publication date of the respective study, and there was no outcome information about 67 patients (n=24.4%) (Table 1). Five studies^[2,4,6,15,18]^ (n=219) reported deaths during treatment, totaling 77 patients (35.2%), among which, two studies^[2,6]^ developed in the People's Republic of China had the highest mortality rate.

**Fig. 2:**
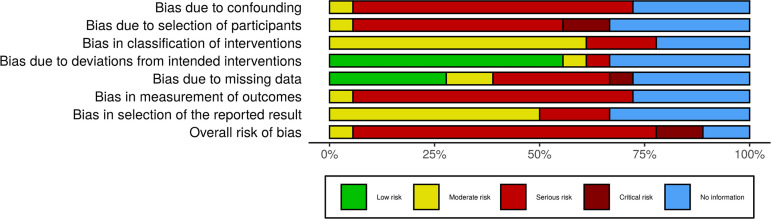
Risk of bias in studies.

When analyzing the risk of bias in different domains studies, it is noticed that there is a predominance of serious risk of bias due to confounding, to selection of participants, and in measurement of outcomes, however, the studies presented low risk of bias due to deviations from the intended interventios and moderate risk on what concerns to bias in classification of interventions and selection of the reported result. Bias due to missing data presented moderate, low, and no information risk proportional between selected papers. The overall risk of bias of all selected articles was classified as serious (Figure 2).

## DISCUSSION

Most patients with COVID-19 have mild symptoms and evolve to cure of the disease. However, some of them progress to a severe state of the disease, developing dyspnoea and hypoxemia about a week after onset. Such patients can rapidly progress to ARDS and, later, to multiple organ failure or even death^[10]^.

WHO and the Centers for Disease Control and Prevention, or CDC, through the publication of guidelines, indicate the possibility of treatment by ECMO in patients in severe/critical condition, with respiratory failure and cardiac involvement, whose conventional treatment is not being promising^[17]^.

ECMO is indicated in patients with refractory hypoxemia with a partial pressure of oxygen (PaO_2_)/fraction of inspired oxygen (FiO_2_) < 50 mmHg for three hours or a PaO_2_/FiO_2_ < 80 mmHg for > 6 hours^[23]^. Severe and refractory hypoxemia is an event associated with mortality in over 95% of patients. In this scenario, conventional mechanical ventilation is not able to promote minimal blood oxygenation compatible with life. At this time, the extracorporeal oxygenation technique (ECMO) can be used until the lungs recover and regain their basic function^[22]^.

In this review, less than half of the analyzed patients used ECMO. In fact, it is an intensive therapy whose priority is given to a specific group of patients: younger patients with a relatively low prevalence of comorbidities and with an acceptable probability of reversing the pulmonary failure typical of these patients^[25]^. Thus, an adequate clinical judgment and an understanding of the risk-benefit relationship are important to identify when ECMO may be effective^[13]^.

In addition, some factors must be observed, such as old age, comorbidities that portend a poor prognosis (diabetes, heart disease, obesity, among others), and, especially, if patients have hemorrhage in the central nervous system, underlying terminal diseases, or evidence of multisystem organ failure^[5]^.

A fact verified in this study was that the majority of patients who needed ECMO were submitted to the venous type. In fact, except for VA-ECMO cannulation in emergency situations, as in the case of cardiopulmonary resuscitation, patients are placed on VV-ECMO, in order to correct the hypoxia resulting from the lung failure caused by the virus, having considered that the low oxygen content could progress quickly, leading to multiple organ failure^[6]^.

Some patients, during ECMO, may receive antivirals, antibacterial agents, steroids, immunoglobulins, chloroquine, vasoconstrictor agents, or even other medications as complementary treatment. Other concomitant therapies are also possible, such as renal replacement^[20,18]^. Everything will depend on what problems the patient may present as well as the choice of which other treatment will be ideal to help in the recovery of the patient.

It is noteworthy that the role of ECMO in the treatment of the disease caused by this new virus remains uncertain and, in the meantime, new research by several authors is always suggested^[2,5,13,18,19]^. From this perspective, the position among researchers may be controversial, because while some authors tend to be more pessimistic when observing high mortality rates with this type of treatment^[2,19]^, including reporting septic shock and multiple organ failure^[2]^, others suggest that it may play an important role and aid to those in the critical state of ARDS due to COVID-19^[4,6,11]^. Such facts become more evident when assessing the risk of bias in studies.

It should also be noted that the therapeutic modality of extracorporeal ventilation is still not widespread. This scenario may be due to the fact that ECMO is an expensive technology that consumes many resources, which may make it impossible for several countries affected by COVID-19 to pay for it^[6]^. Another important point is that it must be carried out in experienced centers, with qualified professionals, and a multidisciplinary approach^[6,25]^.

### Limitations

The main limitation of this study was the small number of papers that addressed the use of ECMO treatment in the current pandemic.

## CONCLUSION

Based on the above, it is evident that more studies, covering larger populations, should be carried out with regard to the use of ECMO in COVID-19 patients, mainly because it is an alternative to the conventional failed treatment of some critical patients. It can also be seen that with new studies, the mechanisms that involve the disease and death of patients due to COVID-19 could be better evidenced, mainly in critical condition, in order to elucidate the role of ECMO in the treatment of patients affected by COVID-19.

**Table:** 

Authors' roles & responsibilities	
TFO	Substantial contributions to the conception or design of the work; or the acquisition, analysis, or interpretation of data for the work; drafting the work or revising it critically for important intellectual content; final approval of the version to be published
CAOR	Substantial contributions to the conception or design of the work; or the acquisition, analysis, or interpretation of data for the work; drafting the work or revising it critically for important intellectual content; final approval of the version to be published
AGGS	Substantial contributions to the conception or design of the work; or the acquisition, analysis, or interpretation of data for the work; drafting the work or revising it critically for important intellectual content; final approval of the version to be published
LCFSJ	Substantial contributions to the acquisition, analysis, or interpretation of data for the work; translation and critical review of the work; final approval of the version to be published
SHSA	Contributions to the acquisition, analysis, or interpretation of data for the work; revising the work; final approval of the version to be published
EJOC	Contributions to the acquisition, analysis, or interpretation of data for the work; revising the work; final approval of the version to be published
RCA	Contributions to the acquisition, analysis, or interpretation of data for the work; revising the work; final approval of the version to be published
RRM	Contributions to the acquisition, analysis, or interpretation of data for the work; revising the work; final approval of the version to be published
GMA	Contributions to the acquisition, analysis, or interpretation of data for the work; revising the work; final approval of the version to be published
FMSS	Contributions to the acquisition, analysis, or interpretation of data for the work; revising the work; final approval of the version to be published
ERSF	Contributions to the acquisition, analysis, or interpretation of data for the work; revising the work; final approval of the version to be published
CDFS	Substantial contributions to the conception or design of the work; or the acquisition, analysis, or interpretation of data for the work; drafting the work or revising it critically for important intellectual contetn; final approval of the version to be published
